# Plasma neutrophil gelatinase-associated lipocalin and factors related to acute kidney injury and mortality in critically ill cancer patients

**DOI:** 10.3332/ecancer.2019.903

**Published:** 2019-02-14

**Authors:** Bertha M Córdova-Sánchez, Erika B Ruiz-García, Alicia López-Yañez, Mireya Barragan-Dessavre, Andoreni R Bautista-Ocampo, Abelardo Meneses-García, Angel Herrera-Gómez, Silvio A Ñamendys-Silva

**Affiliations:** 1 Department of Critical Care Medicine, Instituto Nacional de Cancerología, Mexico City 14080, Mexico; 2 Translational Medicine Research Laboratory, Instituto Nacional de Cancerologia, Ciudad de Mexico 14080, Mexico; 3 Department of Critical Care Medicine, Fundación Clínica Médica Sur, Mexico City 14050, Mexico; 4 Department of Critical Care Medicine, Instituto Nacional de Ciencias Médicas y Nutrición Salvador Zubirán, Mexico City 14000, Mexico

**Keywords:** plasma neutrophil gelatinase-associated lipocalin, acute kidney injury, critically ill cancer patients, intensive care, critical care

## Abstract

**Rationale:**

Acute kidney injury (AKI) is a frequent complication in critically ill cancer patients.

**Objectives:**

To assess plasma neutrophil gelatinase-associated lipocalin (NGAL) levels and risks factors associated with AKI and mortality.

**Methods:**

We recruited 96 critically ill cancer patients and followed them prospectively. Plasma NGAL levels were determined at intensive care unit (ICU) admission and at 48 hours. We generated receiver operating characteristic curves to assess the ability of NGAL to predict AKI. Logistic regression analysis was performed to determine risks factors associated with AKI. Cox-regression analysis was performed to evaluate 6-month mortality.

**Measurements and main results:**

From 96 patients, 60 (63%) developed AKI and 33 (55%) were classified as stages 2 and 3. In patients without AKI at admission, plasma NGAL levels revealed an area under the curve (AUC) = 0.522 for all AKI stages and an AUC = 0.573 for stages 2 and 3 AKI (85% sensitivity and 67% specificity for a 50.66 ng/mL cutoff). We identified sequential organ failure assessment (SOFA) score (without renal parameters) at admission as an independent factor for developing stages 2 and 3 AKI, and haemoglobin as a protective factor. We observed that metastatic disease, dobutamine use and stage 3 AKI were independent factors associated with 6-month mortality.

**Conclusions:**

In our cohort of critically ill cancer patients, NGAL did not predict AKI. SOFA score was a risk factor for developing AKI, and haemoglobin level was a protective factor for developing AKI. The independent factors associated with 6-month mortality included metastatic disease, dobutamine use, lactate and stage 3 AKI.

## Introduction

Acute kidney injury (AKI) is a common complication in cancer patients due to exposure to contrast media, cytotoxic agents, surgical treatment, antibiotics and nonsteroidal anti-inflammatory drugs. In addition, critically ill cancer patients are at increased risk for AKI associated with volume depletion and sepsis [1].

Studies including critically ill patients with hematologic and solid neoplasms have identified an incidence of AKI ranging from 22 to 100%, using the criteria proposed by the Acute Kidney Injury Network and the organisation Kidney disease: Improving Global Outcomes (KDIGO), additionally the presence of AKI is associated with higher mortality [2–4].

Serum creatinine (SCr) and urinary output (UO) are traditional markers of AKI; however, decreased muscle mass, inflammation, volume expansion or medications can alter SCr production, limiting its sensitivity. Therefore, biomarkers that are more sensitive are under investigation [1].

Plasmatic neutrophil gelatinase-associated lipocalin (NGAL) has demonstrated good predictive performance in children [5]. Nevertheless, in adult critically ill patients, the results are heterogeneous, with an area under the curve (AUC) of less than 0.7 in recent studies [6, 7].

Most studies investigating AKI biomarkers in cancer patients focus on early detection of nephrotoxicity mediated by anticancer drugs [8, 9]. However, in critically ill cancer patients, inflammatory biomarkers, such as NGAL, may be influenced by numerous sources of inflammation and do not reflect tubular damage. Therefore, we aim to explore the predisposing factors of AKI and the performance of NGAL in a population of critically ill cancer patients.

## Methods

**Figure 1.**
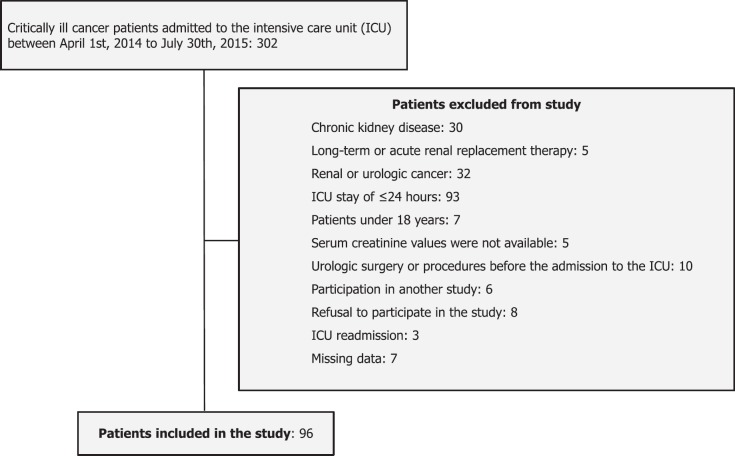
Cohort selection flow diagram.

We performed a prospective observational study from April 2014 to July 2015, at the Instituto Nacional de Cancerología, a tertiary care cancer centre. The institutional ethics committee approved this study (Prot. No. 014/011/SCI/CEI/860). Written informed consent was obtained from all participants or primary caregivers. We included 96 critically ill cancer patients at the time of admission to the ICU. We excluded patients who had an ICU stay of ≤24 hours or patients under 18 years, with known chronic kidney disease (CKD), long-term or acute renal replacement therapy (RRT), previous kidney transplantation and renal or urologic cancer (Figure 1). ICU readmissions were not considered. We estimated glomerular filtration rate (GFR) using the CKD Epidemiology Collaboration equation, CKD was defined as GFR < 60 mL/min/1.73m^2^ for at least 3 months prior to admission [10].

Researchers also recorded demographic characteristics, comorbidities, cancer status, sepsis, mechanical ventilation, acute physiology and chronic health evaluation (APACHE II) [11], sequential organ failure assessment (SOFA) [12] score and laboratory values. In addition, registered days with mechanical ventilation, days with norepinephrine ICU stay and death at 180 days were recorded.

## Acute kidney injury criteria

We defined AKI according to KDIGO Clinical Practice Guidelines. Stage 1 AKI was defined as 0.3 mg/dL SCr elevation above baseline within 48 hours, and/or UO less than 0.5 mL/kg/h for 6–12 hours. Stage 2 AKI was defined as SCr with a 2–2.9-fold increase compared with the baseline value, and/or UO less than 0.5 mL/kg/h for 12–24 hours. Stage 3 AKI was defined as SCr greater than three-fold the baseline value, UO less than 0.3 mL/kg/h for greater than 24 hours, anuria for greater than 12 hours or RRT requirement [13].

We considered baseline creatinine as the lowest value in the 3 months preceding hospitalisation. In patients without historical values, the baseline was calculated by estimating the eGFR at 75 mL/min/1.73 m^2^ [13].

## Plasma collection and processing

Plasma concentrations of NGAL were measured at 24 hours and 48 hours during the stay in the ICU. Plasma samples were freshly collected in tubes with heparin, placed on ice and centrifuged for 15 minutes. The supernatant was stored at −20°C until assayed. Plasma samples were diluted 20 times, with a calibrator included in the commercial kit *Human Lipocalin kit-1/NGAL (R&D Systems)*. The conjugated sample remained stored at 2°C−8°C. The other reagents were left at room temperature before use. The washing buffer was incubated at room temperature and gently stirred until the crystals dissolved. We diluted 20 mL with deionised water to prepare 500 mL of washing buffer. Twenty millilitres of the calibrator RD5-24 were diluted in 80 mL of deionised water to prepare 100 mL solution. The coloured reagents A and B were mixed in equal volumes 15 minutes before use and protected from light. The lipocalin-2 standard was reconstituted with 1 mL of deionised water and incubated for 15 minutes at room temperature. Subsequently, 100 μL of the standard and 900 μL were used for the standard curve. Samples and standards were performed in triplicate.

We used the software *elisaanalysis.com* to perform the analysis and curve adjustment with four logistic parameters. W obtained *r*^2^ = 0.9967093.

## Statistical methods

Continuous variables are expressed as the mean (± standard deviation) or median (interquartile range 25%–75%). Categorical variables are expressed as proportions. To compare patients with and without AKI, we performed Student’s *t*-test or Mann-Whitney *U* test for continuous variables, as appropriate, and X^2^ test was employed for categorical variables. We constructed receiver operating characteristic (ROC) curves to estimate the sensitivity and specificity of NGAL to predict AKI within 72 hours. We performed a logistic regression analysis to determine risks factors associated with AKI, as defined by KDIGO during hospitalisation (expressed as odds ratio and 95% confidence interval: OR, 95% CI). We constructed a univariate Cox-regression analysis to assess factors related to time to death (expressed as hazard ratio: HR). Variables with *p* values <0.05 were included in multivariate analysis.

## Results

### Acute kidney injury

We included 96 critically ill cancer patients. Specifically, 60 patients (63%) developed AKI and 33 (55%) were classified as stages 2 and 3. None of these patients required RRT. Comparison of general characteristics between patients with AKI and patients without AKI is presented in Table 1.

In total, 28 patients already had AKI at ICU admission and 32 developed AKI during their ICU stay. From the 68 patients without AKI at admission, 32 (47%) developed AKI: 19 (28%) were stage 1 patients, 10 (15%) were stage 2 patients and 3 (4%) were stage 3 patients.

**Figure 2.**
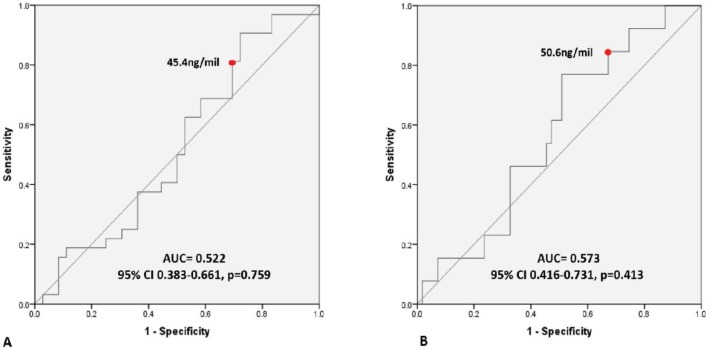
(A) Plasmatic NGAL levels in patients at any stage based on KDIGO criteria. (B) Patients meeting the KDIGO criteria for stages 2 and 3.

In 68 patients without AKI at admission, plasma NGAL levels exhibited an AUC = 0.522 (95% CI: 0.383–0.661, *p* = 0.759) for all AKI stages, with an 81.3% sensitivity and 69.5% specificity for a 45.4 ng/mL cutoff. We observed an AUC = 0.573 (95% CI: 0.416–0.731, *p* = 0.413) for stages 2 and 3 AKI with an 84.6% sensitivity and 67.3% specificity for a 50.66 ng/mL cutoff (Figure 2).

The OR of AKI decreases depending on the increase in haemoglobin level, therefore, we constructed a ROC curve to identify the haemoglobin threshold related to a lesser AKI risk, resulting in ≥ 8.8 g/dL. Then, we categorised patients into two groups and observed that patients with haemoglobin level ≥ 8.8 g/dL had decreased risk of AKI (OR 0.138, 95% CI: 0.036–0.521, *p* = 0.004).

We identified the SOFA score (without renal parameters) at admission as an independent factor (OR 1.30, 95% CI: 1.00–1.68, *p* = 0.049) for developing stages 2 and 3 AKI. Male gender exhibited a trend towards significance (OR 4.6, 95% CI: 0.94–22.12, *p* = 0.06), and haemoglobin appeared as a protective factor (OR 0.76, 95% CI: 0.42–0.94, *p* = 0.026) (Table 2).

We assessed variables for collinearity and interactions and constructed a multivariate model. The AUC of the model was 0.892 (95% CI: 0.807–0.978, *p* = 0.000) for predict stages 2 and 3 AKI and the *p*-value for Hosmer-Lemeshow test was 0.822.

### Factors associated with 6-month mortality

We included 96 patients in mortality analysis (Table 3). We founded that age (HR 1.03, 95% CI: 1.00–1.05, *p* = 0.043), metastatic disease (HR 4.04, 95% CI: 1.45–11.29, *p* = 0.008), dobutamine use (HR 21.92, 95% CI: 3.68–130.5, *p* = 0.001), lactate at admission (HR 1.03, 95% CI: 1.01–1.05, *p* = 0.003) and stage 3 AKI (HR 2.83, 95% CI: 1.07–7.48, *p* = 0.036) were independent factors for 6-month mortality. In multivariate analysis, we observed that NGAL measured at 48 hours of admission had an HR = 1.047 (95% CI: 0.993–1.104, *p* = 0.087) for each 10 ng/mL that was not statistically significant to statistical significance to predict 6-month mortality.

The value of haemoglobin at the time of ICU admission demonstrated statistical significance as a protective factor for mortality (HR 0.81, 95% CI: 0.66–0.99, *p* = 0.047).

## Discussion

During the recruitment period, we observed an AKI prevalence of 63%, corresponding with other studies in critically ill oncology patients using definitions such as AKIN and KDIGO [2, 3].

In our group of cancer patients without AKI at the time of admission to the ICU, plasma NGAL displayed a low diagnostic performance with an AUC of 0.522 for all AKI stages. Plasma NGAL also predicted moderate to severe AKI with an AUC of 0.573 for stages 2 and 3. This finding is consistent with various previous studies in critically ill adult patients, in whom the diagnostic performance of NGAL is more variable compared with paediatric patients [5, 14] and adult patients exposed to contrast media or cardiac surgery [15].

Studies designed to assess the diagnostic performance of NGAL to predict AKI in critically ill patients have revealed a low AUC ranging from 0.44 to 0.7 for plasma NGAL [6, 7, 16] and values from 0.5 to 0.8 for urinary NGAL [7, 16–18].

Generally, these studies compared the diagnostic accuracy of biomarkers with SCr level and did not employ precise methods, such as renal scintigraphy, to verify the reduction in glomerular filtration [15]. The results can be conflicting in ICU patients, given that hypercatabolism, malnutrition, fluid overload and drugs that affect tubular excretion can modify SCr [19, 20].

On the other hand, biomarkers utility depends on sample timing after renal insult. However, in ICU patients, AKI is frequently multifactorial and it is not possible to determine the exact moment of the renal insult [21].

Moreover, the burden of comorbidities, such as cardiovascular and renal disease may be the cause of the reported difference in NGAL performance between adults and children [18]. Additionally, oncology patients possess factors that could alter NGAL expressions, such as exposure to chronic inflammation and anti-neoplastic drugs that accumulate in the renal cortex and predispose to kidney damage before acute insult [22, 23].

Recently, novel biomarkers of kidney injury have shown promising results to predict AKI but these biomarkers are prone to fail in a heterogeneous population of adult patients. Therefore, it is necessary to use predictive models that consider epidemiological and clinical factors [24, 25]. We found that SOFA score at admission may be useful for predicting the development of AKI and that haemoglobin level is a protective factor. This finding is consistent with previous studies demonstrating that AKI in critically ill cancer patients is associated with multiple organ failures [26], and anaemia is a risk factor for AKI in general ICU patients [24].

Han *et al* [27] previously demonstrated that anaemia defined by haemoglobin < 10.5 g/dL is an independent risk factor for AKI and mortality in a large cohort of critically ill patients, including less than 18% with neoplastic disease. In our population, we observed a mean haemoglobin level of 9.6 g/dL and a threshold of 8.8 g/dL as a risk factor for AKI. This lower haemoglobin level could be related to cytopenia in oncology patients.

Critically ill patients are exposed to hypoperfusion, and the presence of anaemia decreases the delivery of oxygen to tissues, including renal parenchyma, which normally receives 20%–25% of cardiac output. Additionally, we observed that a higher level of haemoglobin relates to lower mortality rates. The relation between anaemia and mortality occurs because anaemia can aggravate organic dysfunctions [27].

In a six-month mortality analysis, we identified independent factors, such as age, metastatic disease, and severe AKI, previously described in this group of patients [28, 29]. We also identified that the use of dobutamine was statistically significant. One possible explanation is that almost one-third of our patients were septic, and actual guidelines for sepsis management suggest the use of dobutamine in the presence of myocardial dysfunction manifested by elevated cardiac filling pressures, low cardiac output, and signs of hypoperfusion, which may reflect illness severity. However, dobutamine increases cardiac output and splanchnic blood flow. In addition, dobutamine has not demonstrated a benefit in mortality and could even decrease survival [30, 31].

Based on the inclusion of SOFA score and haemoglobin in a risk model without considering renal biomarkers, we identified an AUC = 0.892. Although we obtained this result in a small cohort of patients, these findings can be useful in the construction of future models in critically ill cancer patients, for further validation in larger cohorts. We observed that NGAL measured at 48 hours of admission had an HR = 1.047 (95% CI: 0.993−1.104 for each 10 ng/mL, *p*-value = 0.087) that was not statistically significant to predict 6-month mortality. Increased NGAL levels at 48 hours could be a manifestation of the persistence of inflammation independent of kidney function [32–34].

Our study has several limitations, such as the number of patients, the heterogeneity of the population and the inability to determine the precise moment at which the renal insult occurred in a cohort of medical and surgical critically ill patients. However, most studies about renal biomarkers in cancer patients evaluate their ability to predict AKI in haemodynamically stable patients who receive nephrotoxic antineoplastic drugs. Currently, cancer patients are more frequently admitted to ICUs; therefore, exploration of the diagnostic performance of new instruments is required to improve patient care.

## Conclusion

In our cohort of critically ill cancer patients, NGAL did not predict AKI. SOFA score was a risk factor for developing AKI, and haemoglobin level was a protective factor for developing AKI. The independent factors associated with 6-month mortality included metastatic disease, dobutamine use, lactate and stage 3 AKI.

## Author contributions

Bertha M. Córdova-Sánchez, Silvio A. Ñamendys-Silva had full access to all data used in the study and take responsibility for the integrity of the data and the accuracy of the data analysis. Data responsible: Erika B. Ruiz-García, Alicia López-Yañez, Mireya Barragan-Dessavre, Andoreni R. Bautista-Ocampo, Abelardo Meneses-García, Angel Herrera-Gómez. Study concept and design: Silvio A. Ñamendys-Silva. Statistical analysis: Bertha M. Córdova-Sánchez, and Silvio A. Ñamendys-Silva. Interpretation of data: All authors. Drafting of the manuscript: Bertha M. Córdova-Sánchez, and Silvio A. Ñamendys-Silva. Critical revision of the manuscript for important intellectual content: All authors. Obtained funding: Abelardo Meneses-García, Angel Herrera-Gómez, Erika B. Ruiz-García, and Silvio A. Ñamendys-Silva. Study supervision: Silvio A. Ñamendys-Silva.

## Scientific knowledge on the subject

AKI is a frequent complication in ICU patients. Studies designed to analyze the ability of NGAL to predict AKI in ICU have shown variable results. Oncological patients exhibit factors that can alter the expression of NGAL. However, studies of biomarkers in oncological patients mostly include haemodynamically stable patients exposed to cytotoxic agents.

## What this study adds to the field

This study demonstrates that in critically ill cancer patients the plasma NGAL was not a good predictor of AKI. We identified SOFA score at admission as a risk factor and haemoglobin level as a protective factor for developing AKI.

## Conflicts of interest

None of the authors has a financial relationship with a commercial entity that has an interest in the subject of this manuscript.

## Funding

None.

## Notes

This study was presented in part at the The American Thoracic Society 2018 International Conference, 18-23 May 2018 in San Diego, CA, USA.

## Figures and Tables

**Table 1.** Clinical characteristics of patients on admission to the ICU.

Variable	No AKI (*n* = 36)	AKI (*n* = 60)	*P* value
Male gender, *n* (%)	14 (38.9)	27 (45)	0.558
Age, mean (±SD)	45 (16)	50 (15)	0.159
Oncologic disease			
Haematologic malignancy, *n* (%)	6 (17)	23 (38)	0.025*
Solid tumour *n* (%)	30 (83)	37 (62)	
Previous chemotherapy, *n* (%)	22 (61)	39 (65)	0.720
Metastatic disease, *n* (%)	16 (44)	21 (35)	0.357
ECOG, median (Q1, Q3)	1 (1−2)	1 (1−2)	0.847
Comorbidities			
Type 2 diabetes mellitus, *n* (%)	3 (8)	14 (23)	0.062^†^
Systemic hypertension, *n* (%)	4 (11)	18 (30)	0.033*
Human Immunodeficiency Virus infection, *n* (%)	0 (0)	2 (3)	0.268
ICU admission characteristics			
Postoperative care, *n* (%)	25 (69)	21 (35)	0.001*
APACHE II score, median (Q1, Q3)	13 (8−16)	15 (12−21)	0.005*
SOFA score, median (Q1, Q3)	4 (2−7)	7 (4−9)	0.004*
Mechanical ventilation, *n* (%)	19 (53)	37 (62)	0.392
Sepsis, *n* (%)	6 (17)	23 (38)	0.025*
Norepinephrine, *n* (%)	14 (39)	40 (67)	0.008*
Dobutamine, *n* (%)	0 (0)	3 (5)	0.289
Cardiac arrhythmias, *n* (%)	0 (0)	6 (10)	0.050^†^
Sodium, median (Q1, Q3), (mEq/L)	138 (134−141)	138 (135−140)	0.914
Potassium, median (Q1, Q3), (mEq/L)	3.8 (3.4−4.1)	3.9 (3.4−4.8)	0.173
Haemoglobin, mean (±SD), (g/dL)	10.2 (2.45)	9.3 (2.38)	0.065^†^
Leucocytes, median (Q1, Q3), (×10^9^/L)	10.6 (5−13.6)	7.1 (1.2−14.7)	0.264
Neutrophils, median (Q1, Q3), (×10^9^/L)	9.5 (4.6−12.3)	6.1 (1.6−13.2)	0.292
Platelets, median (Q1, Q3), 10^9^/L	151 (105−231)	176 (57−227)	0.588
Bilirubin, median (Q1, Q3), (mg/dL)	0.9 (0.65−1.83)	0.8 (0.5−1.3)	0.193
pH, median (Q1, Q3)	7.33 (7.28−7.41)	7.38 (7.26−7.43)	0.375
Lactate, median (Q1, Q3), (mmol/L)	2.1 (1.6−3.3)	2.5 (1.6−4.7)	0.366
Renal function			
Basal creatinine, mean (±SD), (mg/dL)	0.75 (0.17)	0.73 (0.19)	0.743
Basal CKD-EPI, mean (±SD)	104 (21)	101 (20)	0.512
Creatinine at ICU admission, median (Q1, Q3), (mg/dL)	0.67 (0.58−0.76)	0.93 (0.67−1.4)	<0.001*
BUN at admission, median (Q1, Q3), (mg/dL)	10.7 (6.9−17.3)	17 (11.1−24.5)	0.003*
NGAL at admission, median (Q1, Q3), (ng/mL)	95.8 (28−196.8)	77.5 (45.5−138.1)	0.931
NGAL at 48 hours, median (Q1, Q3), (ng/mL)	65.9 (26.9−109.8)	85.9 (34.5−126.6)	0.258
Delta NGAL, median (Q1, Q3), (ng/mL)	−19.6 (−78 to +5)	−11.1 (−39.8 to +14.4)	0.102
Outcomes			
Length of mechanical ventilation, median (Q1, Q3), days	1 (0−5)	1 (0−5)	0.373
Days with norepinephrine, median (Q1, Q3)	0 (0−2)	2 (0−3)	0.026*
Length of stay in ICU, median (Q1, Q3), days	2 (1−4)	3 (1−5)	0.198
Death at ICU, *n* (%)	5 (14)	7 (12)	0.750
Death at 180 days, (%)	6 (17)	27 (45)	0.005*

* *p* value < 0.05 trend towards significance.

AKI: Acute kidney injury, ECOG: Eastern Cooperative

Oncology Group, ICU: intensive care unit, APACHE: Acute physiology and chronic health evaluation, SOFA: Sequential organ failure assessment, CKD-EPI: Chronic Kidney Disease Epidemiology Collaboration, BUN: Blood urea nitrogen, NGAL: neutrophil gelatinase-associated lipocalin.

**Table 2.** Factors associated with AKI.

Variable	Bivariate analysis			Multivariate analysis		
	OR	95% CI	*P* value	OR	95% CI	*P* value
Age	1.019	0.982−1.058	0.315	1.035	0.988−1.083	0.146
Male gender	3.937	1.073−14.44	0.039*	4.552	0.937−22.12	0.060
Haematologic malignancy	4.667	1.304−16.70	0.018*	0.764	0.112−5.209	0.783
Haemoglobin	0.640	0.470−0.873	0.005*	0.626	0.415−0.944	0.026*
SOFA score	1.305	1.068−1.594	0.009*	1.296	1.001−1.678	0.049*

* *p* value < 0.05, SOFA: Sequential organ failure assessment.

**Table 3.** Cox proportional hazards analyses of factors associated with increased 6-month mortality rate.

Variable	Bivariate analysis			Multivariate analysis		
	HR	95% CI	*P* value	HR	95% CI	*P* value
Age	1.017	0.994−1.040	0.151	1.025	1.001−1.050	0.043*
Male gender	1.017	0.994−1.040	0.151	0.763	0.356−1.635	0.487
Postoperative care	0.470	0.218−1.013	0.054	0.657	0.254−1.701	0.387
Metastatic disease	2.280	1.083−4.802	0.030*	4.044	1.448−11.29	0.008*
Human Immunodeficiency Virus infection	5.189	1.164−23.13	0.031*	4.857	0.797−29.62	0.087
Dobutamine	8.079	1.736−37.61	0.008*	21.92	3.682−130.5	0.001*
Haemoglobin	0.883	0.764−1.021	0.093	0.809	0.657−0.997	0.047*
Total neutrophils	0.944	0.888−1.004	0.067	0.973	0.920−1.029	0.337
Lactate at ICU admission	1.024	1.010−1.038	0.001*	1.028	1.010−1.046	0.003*
NGAL at 48 hours (for each 10 ng/mL)	1.046	1.000−1.097	0.061	1.047	0.999−1.104	0.087
KDIGO stage 3	3.520	1.129−10.98	0.030*	2.828	1.069−7.480	0.036*

* *p* value <0.05.

ICU: intensive care unit, NGAL: neutrophil gelatinase-associated lipocalin, KDIGO: Kidney Disease: Improving Global Outcomes.
